# Self-Etching Primer Or Hydrofluoric Acid: Effects On the Bond Strength Stability of a Leucite-Based Glass-Ceramic

**DOI:** 10.3290/j.jad.c_2092

**Published:** 2025-06-11

**Authors:** Camila da Silva Rodrigues, Manassés Tercio Vieira Grangeiro, Rita Adriana Souza da Silva de Assi, Mateus Gaya dos Santos, Marco Antonio Bottino, Renata Marques de Melo

**Affiliations:** a Camila da Silva Rodrigues Professor, Department of Dental Materials and Prosthodontics, Institute of Science and Technology, São Paulo State University. Av. Eng. Francisco José Longo, 777, 12245-000, São José dos Campos, SP, Brazil. Hypothesis, experimental design, performed experiments, wrote the manuscript, performed statistical analyses.; b Manassés Tercio Vieira Grangeiro Professor, School of Dentistry, Anhanguera University. Av. João Batista de Souza Soares, 4009, 12236-660, São José dos Campos, SP, Brazil. Idea, hypothesis, experimental design, performed experiments, proofread the manuscript.; c Rita Adriana Souza da Silva de Assis PhD Candidate, Department of Dental Materials and Prosthodontics, Institute of Science and Technology, São Paulo State University. Av. Eng. Francisco José Longo, 777, 12245-000, São José dos Campos, SP, Brazil. Performed experiments, contributed substantially to discussion, proofread the manuscript.; d Mateus Gaya dos Santos Former dental Student, School of Dentistry, Federal University of Pelotas. Rua Gonçalves Chaves 457, sala 505, 96015-560, Pelotas, RS, Brazil. Performed experiments, contributed substantially to discussion, proofread the manuscript.; e Renata Marques de Melo Professor, Department of Dental Materials and Prosthodontics, Institute of Science and Technology, São Paulo State University. Av. Eng. Francisco José Longo, 777, 12245-000, São José dos Campos, SP, Brazil; Contributed substantially to discussion, proofread the manuscript, supervised the study.; f Marco Antonio Bottino Professor, Department of Dental Materials and Prosthodontics, Institute of Science and Technology, São Paulo State University. Av. Eng. Francisco José Longo, 777, 12245-000, São José dos Campos, SP, Brazil; Contributed substantially to discussion, proofread the manuscript, supervised the study.

**Keywords:** dental ceramics, adhesion, self-etching

## Abstract

**Purpose:**

To evaluate the effect of a self-etching primer on the long-term bond strength stability between a leucite-based glass-ceramic and resin cement, compared to the conventional treatment involving hydrofluoric acid (HF) etching followed by silane application.

**Materials and Methods**: Blocks of a leucite-based glass-ceramic (IPS Empress CAD) were cut into plates and embedded in acrylic resin. Half of the specimens were treated with 5% HF for 60 s and silane application, and the other half was treated with a self-etching primer (Monobond Etch and Prime, MEP). Resin cement cylinders (n = 24) were built onto their surfaces, and the specimens of each group were divided into three subgroups according to the microshear bond strength (µSBS) testing time: baseline, after 10,000 thermocycles, or after 10,000 thermocycles followed by 180 days of immersion in water. Statistical analysis was performed with two-way analysis of variance and Tukey’s tests. Complementary failure mode, contact angle, and scanning electron microscopy analyses were carried out.

**Results:**

MEP groups showed higher bond strength results than HF. HF-treated specimens exhibited a decrease in bond strength after thermocycling and water storage, while MEP-treated specimens maintained similar bond strength values across all aging conditions. Only cohesive failures within the ceramic were observed at baseline. After aging, most HF specimens exhibited adhesive failures. HF etching created more irregularities with apparent deeper defects on the ceramic surface compared to MEP. HF etching produced a lower contact angle between the ceramic surface and the water drop compared to the self-etching primer.

**Conclusion:**

Applying the self-etching primer resulted in higher bond strength stability between leucite-based glass-ceramic and resin cement compared to conventional treatment.

Glass ceramics represent a class of restorative materials that combine translucency and flexural strength, which makes them suitable for a wide range of restorative applications.^3^ Among these materials, leucite-based glass-ceramic stands out for its high esthetic appearance and has been recommended for single-unit anterior and posterior full or partial coverage crowns.^17^ Thus, clinical studies have reported survival rates of 91% over 5 years^11^ for anterior and 97% over 7 years^15^ for posterior restorations.

Adhesive cementation of ceramic restorations is essential to ensure mechanical strength and stress distribution throughout the restoration and dental substrate.^6,28^ Hence, the gold standard for preparing glass-ceramic surfaces involves etching with hydrofluoric acid (HF) according to the manufacturer’s recommendations, followed by the application of a silane coupling agent.^25^ In this procedure, HF creates surface irregularities for micromechanical bonding with resin cements.^24,30^ while the high glass content allows for the chemical coupling of silane–OH groups to the silica on the ceramic surface.^4^ However, the toxicity of hydrofluoric acid poses potential hazardous effects for clinicians, including skin or nail burns and eye injuries.^22^

Aiming to optimize clinical procedures and avoid the use of HF, a self-etching primer was introduced in 2015 (ie, Monobond Etch and Prime (MEP), Ivoclar Vivadent) and has gained popularity over the years.^33^ The application of this product involves rubbing it onto the ceramic surface to remove contaminants. The product then remains on the surface, allowing the ammonium polyfluoride in its composition to react with the ceramic surface and produce a rough etching pattern. Finally, the product is washed off, initiating the reaction between the silane and the ceramic surface, which results in a thin silane layer.^33^ There is substantial literature on the effects of self-etching primers on the adhesion between glass-ceramics and resin cements.^1,5,9,16,20,29,32^ Nevertheless, most studies have focused on lithium disilicate ceramics, with limited research exploring how MEP affects the adhesive behavior of leucite-based glass-ceramics.^9,20,21,29,32^ Among the few aforementioned studies, common aging protocols include 10,000 cycles of thermocycling or water storage, with variations in the test setups.

Given the lack of information regarding leucite-based glass-ceramics and the variations in the available literature, there is a need for studies to confirm or contrast existing results. Additionally, since resin-based materials undergo hydrolytic degradation due to water sorption,^12^ prolonged aging should be considered. Therefore, this study aimed to evaluate the effect of a self-etching primer on the bond strength stability between a leucite-based glass-ceramic and a resin cement, compared to the conventional treatment of hydrofluoric acid etching followed by silane application. The tested hypothesis was that both treatments would produce similar bond strength stability.

## MATERIALS AND METHODS

### Study Design

This in vitro study evaluated two factors: surface treatment (self-etching primer or HF etching followed by silane) and aging (baseline, 10,000 thermocycles, or 10,000 thermocycles followed by 120-day water storage). The primary response variable was microshear bond strength (µSBS). The materials used in this research are described in Table 1.

**Table 1:** Materials used in this study

Material	Commercial brand	Composition*
Leucite-based glass-ceramic	IPS Empress CAD (Ivoclar)	SiO_2_ (60–65 w%), Al_2_O_3_ (16–20 w%), K_2_O (10–14 w%), Na_2_O (3.5–6.5 w%), other oxides (0.5–7.0 w%), and pigments (0.2-1.0 w%)
Hydrofluoric acid	Condac Porcelana (FGM)	5% hydrofluoric acid
Ceramic primer	Monobond N	Alcohol solution of silane methacrylate, phosphoric acid methacrylate and sulphide methacrylate.
Self-etching primer	Monobond etch & prime	Water, alcohol, ammonium-polyfluoride, phosphoric acid methacrylate, silane methacrylate, dipodal silane, colouring agent.
*Manufacturer’s information

### Specimens Preparation

**Fig 1:**
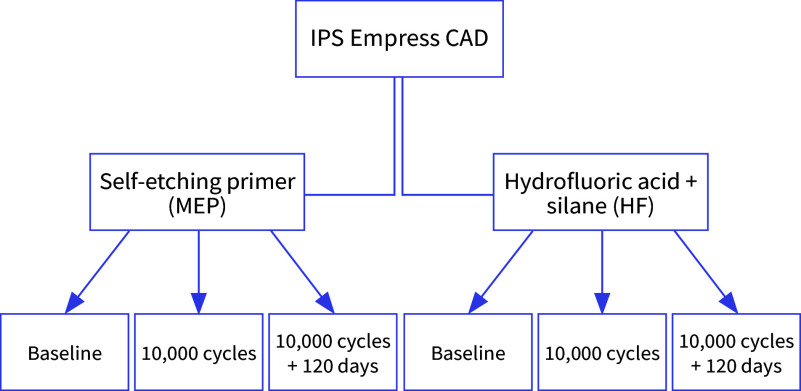
Study design (n = 24).

Blocks of a leucite-based glass-ceramic (IPS Empress CAD, Ivoclar Vivadent, Schaan, Liechtenstein) were cut into plates using a diamond saw under water cooling in a cutting machine (IsoMet 1000, Buehler, Lake Bluff, USA). The ceramic plates were leveled with #400 grit silicon carbide paper, and one surface was polished using a #600 and #1200 grit sequence (Norton Abrasives, Worcester, MA). These procedures were carried out under water cooling in a polishing machine (EcoMet/AutoMet 250, Buehler, Lake Bluff, USA). The specimens were manually held on the polishing machine (200 rpm) using light pressure for 2 min per grit of carbide paper. The carbide papers were changed every four specimens. The specimens with final dimensions of 12 mm × 10 mm × 2 mm were embedded in a chemically activated acrylic resin (JET, Dental Articles Classic, Curitiba, Brazil). To accomplish this, the polished ceramic surfaces were placed down on double-sided sticky tape (Adere, Sumaré, Brazil). Then, 2 cm-high polyvinyl chloride (PVC) cylinders were placed over the tape to encircle each ceramic plate, and the PVC cylinders were filled with acrylic resin. The tape was removed after curing, and the exposed ceramic surfaces were polished for 5 min with #1200 grit carbide paper to ensure complete removal of any sticky tape residue. The specimens were cleaned with ethanol (Ciclo Farma, Serrana, Brazil) in a 5-min ultrasonic bath and randomly divided into six groups, as illustrated in Figure 1. Each group included four ceramic plates, which later received six resin cement cylinders, totaling 24 specimen units per group.

The specimens from the HF groups had the ceramic surfaces with 5% hydrofluoric acid for 60 s (Condac porcelana 5%, FGM Dental Group, Joinville, Brazil). After rinsing off the HF with running water for 60 s, the specimens were ultrasonically cleaned in distilled water for 5 min. Following air-drying, a silane layer (Monobond N silane, Ivoclar Vivadent; Schaan, Liechtenstein) was actively applied to the ceramic surfaces with a microbrush (FGM Dental Group, Joinville, Brazil) for 10 s and left to dry for 60 s. The MEP groups had the self-etching primer rubbed onto the ceramic surfaces with a microbrush for 20 s. After 40 s, the product was rinsed off with running water, and the specimens were subjected to a 5-min ultrasonic bath in distilled water. Cutting, polishing, and surface treatment steps were performed by two previously trained operators.

The resin cement cylinders were built on the treated ceramic surfaces by placing six silicone tubes (internal diameter = 1.75 mm, height = 2 mm) on each specimen. The silicone tubes were fixed with sticky wax (Asfer, São Caetano do Sul, Brazil) and filled with the luting agent (Multilink N, Ivoclar Vivadent; Schaan, Liechtenstein) using endo auto-mixing tips. Excess resin cement was removed with microbrushes, and each silicone cylinder was photopolymerized for 40 s with a light-curing unit (Valo, Ultradent; South Jordan, USA). One previously trained operator carried out the bonding procedures to avoid bias. A sample size of 20 was estimated considering a minimum detectable difference in means of 3.4 MPa, an expected standard deviation of residuals of 4.0, power of 0.8, and α = 0.05. However, considering the possibility of pre-test failures, 24 resin cement cylinders (n = 24) were prepared for each group.

After the bonding procedures, all specimens were immersed in distilled water at 37°C (Fanem, Orion Estufa de cultura 502, São Paulo, Brazil) for 24 h. The silicone tubes were then carefully cut with razor blades and removed along with the wax, leaving only the cylinders made of resin cement. The baseline groups were immediately subjected to the µSBS tests. The TC groups were subjected to thermocycling for 10,000 cycles in a thermocycling machine (Biopdi, termocycle, São Paulo, Brazil). A two-water bath was set at 5 (± 1)ºC and 55 (± 1)ºC with a dwell time of 30 s. The TC+120 groups underwent the same thermocycling protocol and were then stored in distilled water at 37°C for 120 days prior to the µSBS tests.

### Microshear Bond Strength Tests

The microshear bond strength tests were performed in a universal testing machine (DL-1000, EMIC, São José dos Pinhais, Brazil) using a load cell of 50 KgF. An orthodontic wire (Ø = 0.2 mm) was attached to the load cell and placed at the cylinder/ceramic interface. The shear load was applied perpendicular to the interface at a crosshead speed of 0.5 mm/min until failure. Bond strength values were calculated using the equation R = F/ A, where R is the bond strength (MPa), F is the load to failure (N), and A is the interface area (mm^2^). The circular interface area was calculated using the equation A = πr^2^, where π = 3.14 and r is the radius of the resin cement cylinder (0.88 mm).

### Failure Mode Analysis

The tested ceramic surfaces were examined under a stereomicroscope (Stereo Discovery V20, Zeiss, Göttingen, Germany) to determine the failure modes as follows: adhesive – when the failure occurred at the interface between the ceramic and resin cement; predominantly adhesive – when more than 50% of the failure area occurred at the interface between the ceramic and resin cement; cohesive within the resin cement – when more than 50% of the failure area occurred within the resin cement; or cohesive within the ceramic – when more than 50% of the failure area occurred within the ceramic material. Representative specimens of each observed failure mode were analyzed using a scanning electron microscope (JSM-6610LV, Jeol, Tokyo, Japan) at 15 kV and a magnification of 30×.

### Topographic Analysis

Ceramic plates (n = 1) were prepared as previously described and subjected to each surface treatment (HF or MEP). The specimens were ultrasonically cleaned with ethanol, air-dried, and gold-sputtered. Their surfaces were qualitatively examined using a scanning electron microscope (SEM), and images were taken at 15 kV and magnifications of 500× and 3,000×.

### Contact Angle Analysis

Ceramic specimens (n = 2) from each surface treatment (HF or MEP) were analyzed with a goniometer (Attension, Biolin Scientific, Stockholm, Sweden) to observe the formed contact angle. The sessile drop technique was performed at room temperature (25°C), in which a 2 µl drop of distilled water was perpendicularly dropped at the center of the ceramic surface using a syringe. The contact angle between the water drop and the ceramic surface was measured three times after 5 s. The experiment was performed three times. Images were taken with a camera connected to the goniometer.

### Statistical Analysis

The statistical analysis was performed using the SigmaPlot 12.0 software program (Systat Software, San Jose, USA). The normality and homoscedasticity of the microshear bond strength data were verified using the Shapiro–Wilk and Levene’s tests, respectively. Afterwards, a two-way analysis of variance (ANOVA) was carried out, considering the surface treatment and aging factors. Multiple comparisons were performed using Tukey’s test. The significance level was set at 5%.

## RESULTS

### Microshear Bond Strength Analysis

Table 2 presents the microshear bond strength results obtained from each experimental group. Both surface treatment and aging factors significantly affected the results (P <0.001 and P = 0.029, respectively), as did the interaction between these factors (P = 0.033). The self-etching primer led to higher bond strength results than hydrofluoric acid followed by silane application under all tested conditions. In addition, HF-treated specimens exhibited a decrease in bond strength after thermocycling and water storage compared to the baseline tests. In contrast, specimens treated with the self-etching primer maintained similar bond strength values across all aging conditions. One specimen from HF group subjected to thermocycling and water storage did not endure aging and was considered a pre-test failure.

**Table 2:** Means and standard deviations (in MPa) of microshear bond strength obtained from each experimental group

Surface treatment	Baseline	10 k	10 k + 120 days
HF	15.75 (4.61)^Ab^	14.23 (4.33)^ABb^	11.54 (1.58)^Bb*^
MEP	18.63 (4.21)^Aa^	17.37 (3.77)^Aa^	18.39 (4.47)^Aa*^
Different uppercase letters within a row indicate statistical differences among aging conditions for each adhesion protocol. Different lowercase letters within a column indicate statistical differences between the adhesion protocols at each aging condition (Two-way ANOVA, Tukey’s test, P < 0.05). HF: 5% hydrofluoric acid etching followed by silane application. MEP: self-etching primer. *n = 23 due to pre-test failures

### Failure Mode Analysis

**Fig 2:**
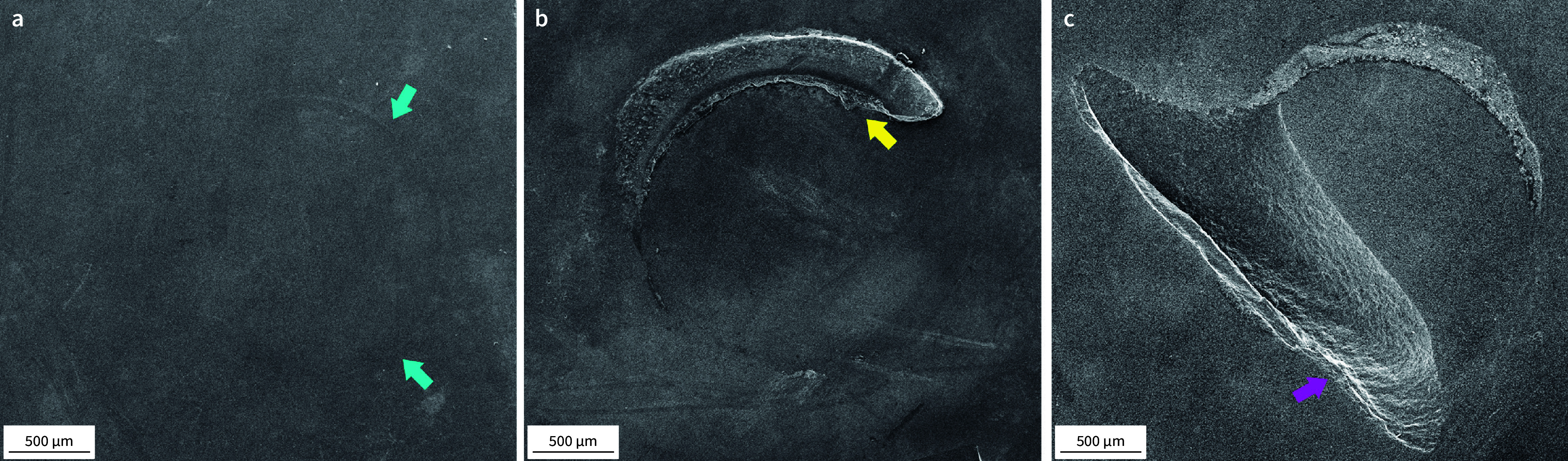
Representative images (30×) of the observed failure modes: (a) adhesive, (b) predominantly adhesive, and (c) cohesive within the ceramic. Blue arrows in (a) indicate the slightly marked area where the resin cement cylinder detached. The yellow arrow in (b) highlights the resin cement remnant from the detached cylinder. The pink arrow in (c) points to the cohesive ceramic fracture that occurred during testing.

**Fig 3:**
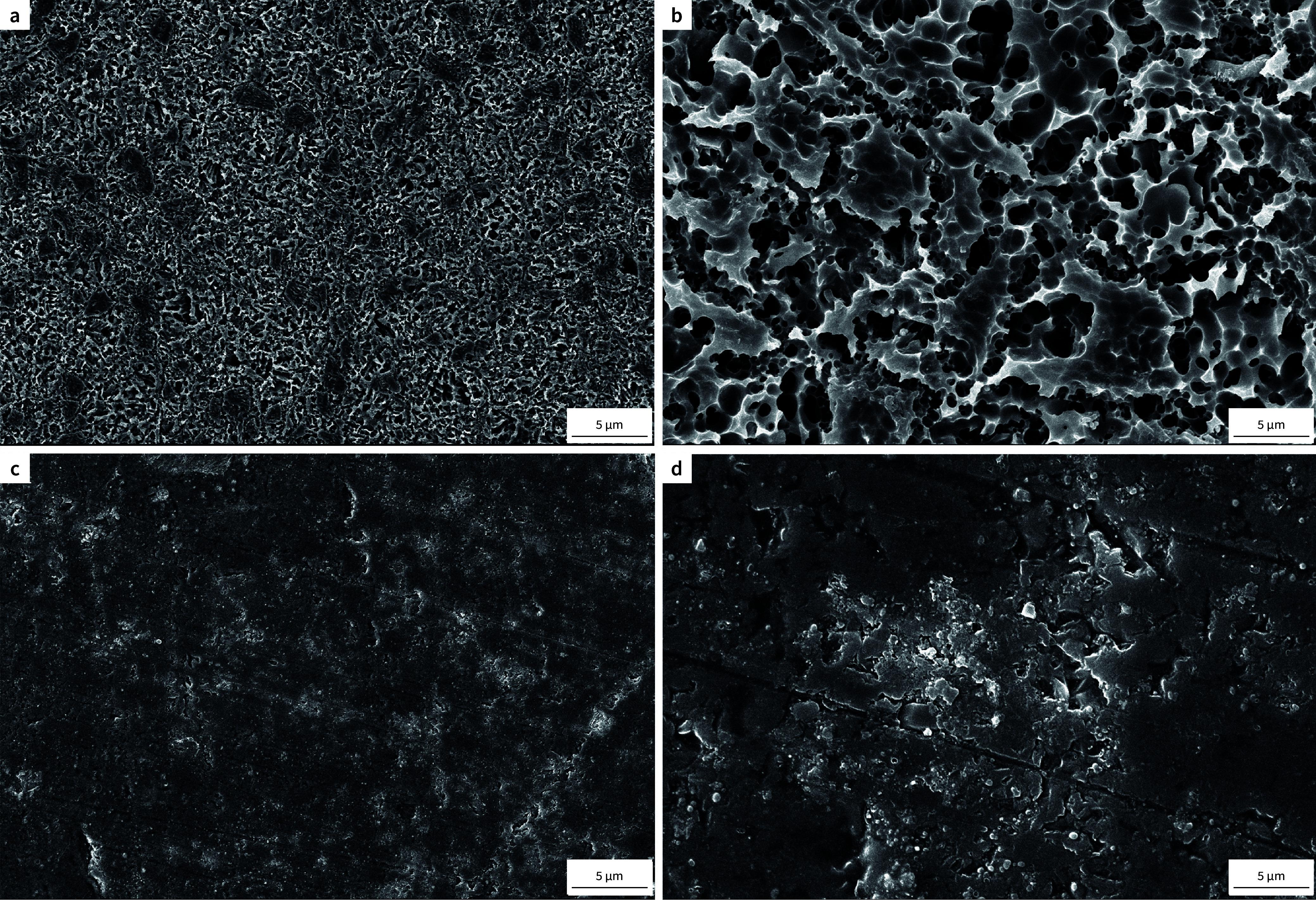
Failure mode distribution among the experimental groups. The most frequent type was cohesive at the ceramic surface, which tended to decrease after aging, especially in the groups with lower bond strength results.

One pre-test failure was observed in both the HF and MEP groups after thermocycling and water storage. Figure 2 illustrates the failure modes observed on the ceramic surfaces in SEM images. The failure mode distribution among the experimental groups is depicted in Figure 3. Both groups exhibited 100% cohesive failures within the ceramic at baseline. Following thermocycling and thermocycling plus water storage, MEP-treated specimens showed 100% and 88% cohesive failures, respectively. After aging, most HF specimens exhibited adhesive failure modes.

### Topographic Analysis

**Fig 4:**
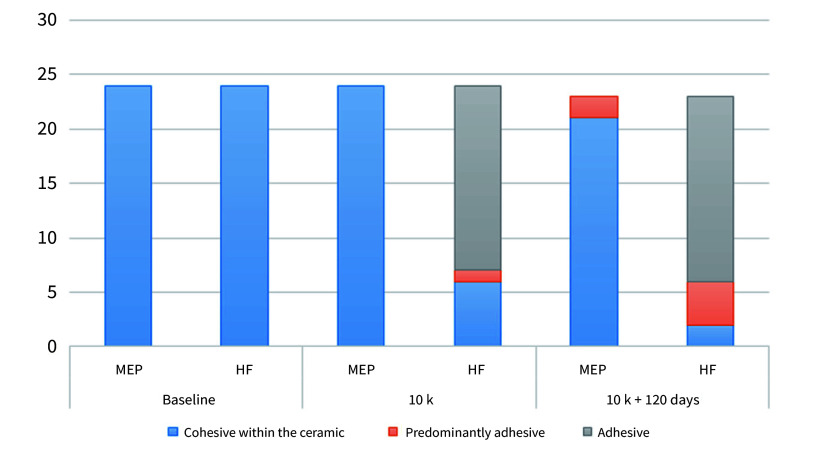
SEM images of leucite-based glass-ceramic after 5% hydrofluoric acid etching (a, b) and after the self-etching primer application (c, d). The images were taken at 500× (a, c) and 3,000× (b, d). More irregularities are observed on the HF-etched surface.

Figure 4 displays the SEM images, which evidences that HF etching created more irregularities with apparent deeper defects on the ceramic surface compared to MEP. Wider and shallower grooves are observed on the surfaces treated with the self-etching primer.

### Contact Angle Analysis

**Fig 5:**
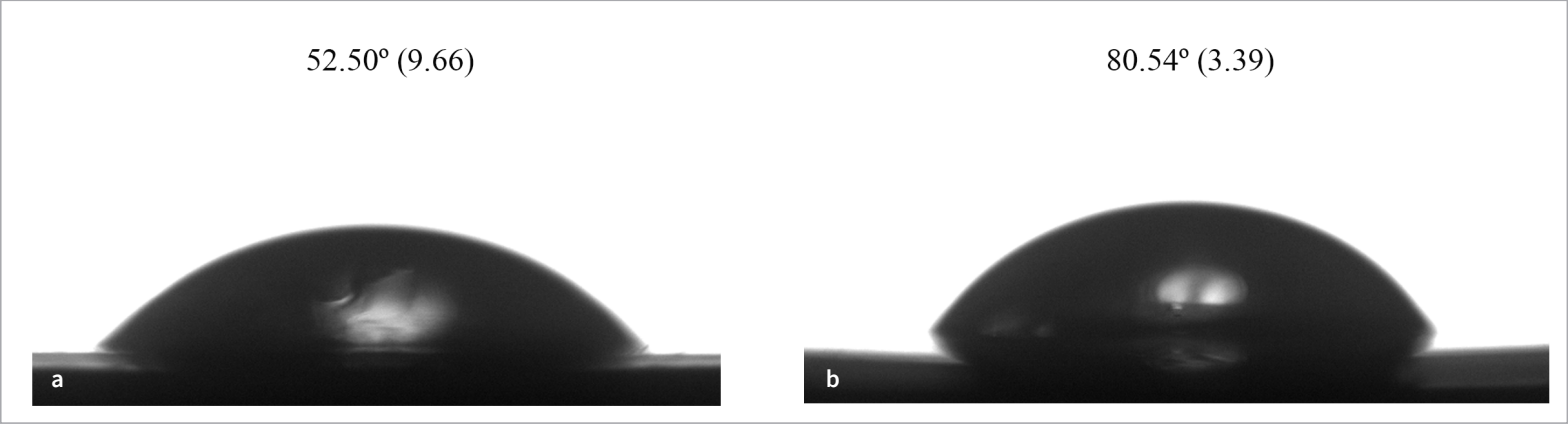
Images and contact angle mean and standard deviation values obtained from the leucite-based glass-ceramic treated with 5% HF for 60 s (a) and with the self-etching primer (b). Lower contact angle was produced after HF etching.

Figure 5 describes the images and mean values acquired from the contact angle analysis. Qualitative analysis demonstrated that the HF etching produced a lower contact angle between the ceramic surface and the water drop compared to the self-etching primer.

## DISCUSSION

The self-etching primer application led to higher microshear bond strength values compared to hydrofluoric acid etching followed by silane application under all tested conditions. Additionally, MEP maintained bond strength stability after aging, whereas the conventional treatment showed a significant decrease after thermocycling and water storage. Therefore, the tested study hypothesis was rejected.

Our SEM images revealed a more irregular surface pattern when the ceramic was treated with hydrofluoric acid (Fig 4). The greater irregularities on glass-ceramic surfaces caused by HF compared to MEP have been demonstrated in several studies.^7,9,27,32^ In addition, the greater surface defects caused by HF acid etching led to a lower contact angle than MEP, as evidenced in Figure 5. A lower contact angle is usually associated with higher bond strength.^26^ However, the shallower defects created by MEP on the leucite-based glass-ceramic surface may have facilitated the luting agent penetration into the irregularities. In contrast, despite the lower contact angle, the deeper defects caused by HF may not have been completely filled, leaving gaps that acted as stress concentrators and made debonding easier to propagate. In addition, these defects may have promoted water sorption, leading to bond strength instability in HF-etched specimens. The easier filling of shallower defects caused by MEP has been illustrated in a previous study using a vitreous ceramic with a similar composition to the one used in this study (ie, feldspathic ceramic).^7^ The authors reported cross-section images that revealed interface defects, especially when using HF. Thus, the surface defect pattern caused by MEP on the tested glass-ceramic seems to play a crucial role in proper filling by the resin cement, resulting in high bond strength both initially and after long-term aging.

Besides the role of surface defects on the bond strength results, bond degradation over time remains a concern with silane-based coupling agents, as these molecules are highly unstable and have an inherent tendency to hydrolyze in water.^8,19^ Previous studies have reported lower bond strength between glass-ceramics and resin cements after aging, attributed to interface degradation.^13,20,29^ The failure mode analysis demonstrated an increase in adhesive failures in HF-etched specimens after undergoing the tested aging conditions, which may be associated with degradation of the resin cement/glass-ceramic interface in water. The occurrence of more adhesive failures after aging and the observation of more cohesive failures in groups with higher bond strength have been described in previous studies with similar designs to ours.^13,26^

Several articles report better bonding behavior of HF and silane compared to MEP. However, one should note that the majority of these studies focused on lithia-based glass-ceramics.^2,10,18^ When analyzing the published results on feldspathic or leucite-based ceramics, several studies reported that the self-etching primer produces similar or superior bonding behavior.^5,20,21^ Feldspathic and leucite-based ceramics have less crystalline content (~32–35 vol%^3^) than lithia-based glass-ceramics (up to 80 vol%^34^). It has been reported that the tested self-etching primer creates different irregularity patterns depending on the ceramic microstructure.^9,20^ This was evidenced in profilometer and SEM images of a previous study,^9^ where ceramics with more glass content showed a more irregular surface than those with more crystalline content (ie, lithium disilicate) when etched with MEP. The different defect patterns, combined with other bonding factors such as resin cement, likely explain why the self-etching primer may be more effective in some ceramics than in others. Our study used 5% hydrofluoric acid, which is the concentration recommended by the tested dental ceramic manufacturer. Previous research has reported that different acid concentrations can produce different defect patterns.^31^ However, a systematic review demonstrated that using acid concentrations either below or above 5% does not impact the bond strength of leucite-based glass-ceramics, provided that the 60-s etching time is maintained.^25^ Hence, the results of this study would not be different if our control group had used a higher-concentration hydrofluoric acid.

Regarding leucite-based glass-ceramics, one study performed 10,000 thermocycles on all specimens and revealed similar shear bond strength (SBS) in groups treated with HF or MEP.^32^ Similarly, two studies that compared SBS^21^ or micro-tensile bond strength (µTBS)^20^ produced by MEP and HF did not report statistical differences either at baseline or after 1-year water storage. In contrast, one study in which all specimens underwent 5,000 thermocycles and performed SBS,^9^ and another study in which µTBS^29^ was performed initially and after 6 months of water storage, observed inferior results with MEP compared to HF. The aforementioned studies employed a variety of methodologies, resin cement compositions, and commercial brands, with tested sample sizes ranging from 10 to 15, which might explain the different findings. In our study, the µSBS test was chosen due to its lower probability of introducing defects,^14^ given that it is a micro test, and its easier preparation compared to µTBS, which may induce stresses during cutting. Furthermore, we tested 24 interfaces per group to ensure an adequate sample size and low standard deviation. Even though previous literature does not show superior bond strength between leucite-based glass-ceramics and resin cements when MEP is applied, some authors have observed stable values after different aging protocols,^21,29^ which is consistent with our findings.

Our findings indicate that the self-etching primer can be used as an alternative to the conventional treatment of leucite-based glass-ceramics, optimizing clinical steps and providing better stability of the adhesive interface. Nevertheless, one may point out that our specimens were highly polished previously to the surface treatments, which differs from clinical conditions where the cementation surface is rough due to the computer-aided design/computer-aided manufacturing (CAD/CAM) milling process. Previous research has reported that rough surfaces, including those produced by milling, lead to higher bond strength than polished surfaces.^23^ Consequently, the lack of a more realistic surface is a limitation of this study, and future research should include in-lab surface simulations to confirm whether differences between HF and MEP persist. Currently, using the self-etching primer is more expensive than using hydrofluoric acid followed by silane. However, if future studies corroborate our findings, adopting this approach might be beneficial for reducing clinical time and improving the longevity of restorations.

## CONCLUSION

Applying the self-etching primer resulted in higher bond strength between leucite-based glass-ceramic and resin cement compared to hydrofluoric acid etching followed by silane application, both before and after aging. Additionally, a decrease in bond strength was observed in the HF group after thermocycling and water storage. In contrast, the specimens treated with MEP remained stable under all tested aging conditions.

### Clinical Relevance

The self-etching primer enhances the long-term bond strength stability between leucite-based glass-ceramic and resin cement, offering a more reliable adhesive interface than HF etching. This finding suggests a clinically relevant approach to improve the performance of this glass-ceramic.

### Acknowledgments

This study was partially financed by the Brazilian Federal Agency for Coordination of Improvement of Higher Education Personnel (CAPES, finance code 001). The authors are also thankful to the São Paulo Research Foundation (FAPESP, Grant number 2020/15720-2). The authors are also thankful to the São Paulo Research Foundation (FAPESP, Grant numbers 2020/15720-2 and 2023/05223-0).
